# N-terminal domain of the architectural protein CTCF has similar structural organization and ability to self-association in bilaterian organisms

**DOI:** 10.1038/s41598-020-59459-5

**Published:** 2020-02-14

**Authors:** Artem Bonchuk, Sofia Kamalyan, Sofia Mariasina, Konstantin Boyko, Vladimir Popov, Oksana Maksimenko, Pavel Georgiev

**Affiliations:** 1 Center for Precision Genome Editing and Genetic Technologies for Biomedicine, Institute of Gene Biology, Russian Academy of Sciences, 34/5 Vavilov St., Moscow, 119334 Russia; 2 Department of the Control of Genetic Processes, Institute of Gene Biology, Russian Academy of Sciences, 34/5 Vavilov St., Moscow, 119334 Russia; 3 Skolkovo Institute of Science and Technology, Skolkovo, 143025 Russia; 4 Department of Chemistry, M.V. Lomonosov Moscow State University, Moscow, 119991 Russia; 5 Faculty of Fundamental Medicine, Center for Magnetic Tomography and Spectroscopy, M.V. Lomonosov Moscow State University, Moscow, 119991 Russia; 6 Bach Institute of Biochemistry, Research Center of Biotechnology Russian Academy of Sciences, Leninsky pr-t, 33, bld. 2, Moscow, 119071 Russia; 7 National Research Center «Kurchatov Institute», Moscow, Russia

**Keywords:** Chromatin, Protein folding, Transcription

## Abstract

CTCF is the main architectural protein found in most of the examined bilaterian organisms. The cluster of the C2H2 zinc-finger domains involved in recognition of long DNA-binding motif is only part of the protein that is evolutionarily conserved, while the N-terminal domain (NTD) has different sequences. Here, we performed biophysical characterization of CTCF NTDs from various species representing all major phylogenetic clades of higher metazoans. With the exception of Drosophilides, the N-terminal domains of CTCFs show an unstructured organization and absence of folded regions *in vitro*. In contrast, NTDs of *Drosophila melanogaster* and *virilis* CTCFs contain unstructured folded regions that form tetramers and dimers correspondingly *in vitro*. Unexpectedly, most NTDs are able to self-associate in the yeast two-hybrid and co-immunoprecipitation assays. These results suggest that NTDs of CTCFs might contribute to the organization of CTCF-mediated long-distance interactions and chromosomal architecture.

## Introduction

Chromosomes in the genomes of all higher eukaryotes have a highly organized architecture and consist of discrete topologically associated domains (TADs)^1–5^. TADs often also include smaller domains (sub-TADs) that are flanked by short boundary elements or longer regions (inter-TADs) that contain active chromatin and housekeeping genes. In addition, promoters, enhancers, silencers and insulators form a network of specific distance interactions that properly regulate gene transcription^6–9^. Until now, the unresolved question remains how specific distance interactions between remote regulatory elements are established and maintained through the cell cycle^10^.

Currently, the best-characterized protein involved in the organization of chromosome architecture is CTCF, which was initially found as a transcriptional repressor^11^. It is believed that CTCF is the main architectural protein in mammals, which is responsible for the organization of TAD boundaries and distance interactions between enhancers and promoters^12–16^. CTCF was found in most of the higher eukaryotes including all studied bilateral organisms but is absent in yeast and plants^17,18^. Usually CTCFs from different organisms contain the cluster of eleven C2H2 zinc-finger domains (ZF) localized in the central part of the protein. In human CTCF, ZFs from 3 to 7 recognize specific 15 bp consensus^19^. The DNA-binding ZFs are the most evolutionary conserved among CTCFs that bind to similar sites in most higher eukaryotic genomes^20^. Moreover, it was found that even several chromatin domains controlled by CTCF are conserved in distant species^21^. Other ZF domains are usually less conserved and are involved in recognition of additional minor sequences^22^, interaction with specific RNAs^23^ or proteins^24–27^. The N- and C-termini of CTCF do not have structural domains and are not conserved in evolution^28,29^.

CTCF can support distance-selective interactions between its sites, suggesting that protein-protein interactions are possibly involved in organization and maintaining long-range chromatin interactions. However, dimerization domains have not been found in hCTCF. The N-terminal domain of hCTCF was shown to be intrinsically disordered^30^. The current model suggests that the movement of cohesin complexes along chromatin^31^ is blocked by chromatin-bound CTCF protein, which leads to the formation of chromatin loops between CTCF sites in interphase chromosomes^2,32^. *In vitro* studies have shown that the cohesin complex interacts with the C-terminal domain of hCTCF^31^. The Drosophila CTCF homolog, dCTCF, is often associated with TAD boundaries and insulators^33,34^. dCTCF supports distance interactions between the GAL4 activator and the *white* gene reporter in model transgenic lines^35,36^. A novel multimerization domain was described within *Drosophila* CTCF (dCTCF) protein^37^. Deletion of this domain strongly affects the activity of dCTCF.

The existence of the N-terminal dimerization domain in *Drosophila melanogaster* raised the question about the structure of the N-terminal domain in CTCF from other bilaterian organisms. We found that CTCF in *Drosophila virilis* (dvCTCF) also has the dimerization domain. However, in other selected organisms from different bilaterian clades, the N-terminal CTCF domains are intrinsically disordered and unable to form dimers *in vitro*. Unexpectedly, the N-terminal domains from CTCF of human and several other organisms showed self-interaction in the yeast two-hybrid (Y2H) and co-immunoprecipitation assays.

## Results

### Drosophila CTCF N-terminal multimerization domains display stable fold in solution, but lack of secondary structure

Earlier, we described the N-terminal multimerization module between 70 and 163 aa of CTCF protein from *Drosophila melanogaster* (dmCTCF), which is essential for functional activity of the dmCTCF protein, but the first 70 residues contribute to its stability and together with 70–163 aa most likely are parts of the entire protein domain^37^. In *Drosophila* genus, alignment of the N-terminal regions of CTCFs showed a moderate level of homology with a few conserved sequence blocks in the interval of 1–163 aa according to dmCTCF sequence (Fig. S1). A plausible hypothesis is that N-terminal domains of CTCFs from different *Drosophila* species have a similar organization and dimerization activity. To test this possibility, we selected for further study the N-terminal domain (1–144 aa) of CTCF from *Drosophila virilis* (dvCTCF), which has the comparatively low sequence (49%) homology to dmCTCF in 1–163 interval (Fig. S1).

**Figure 1:**
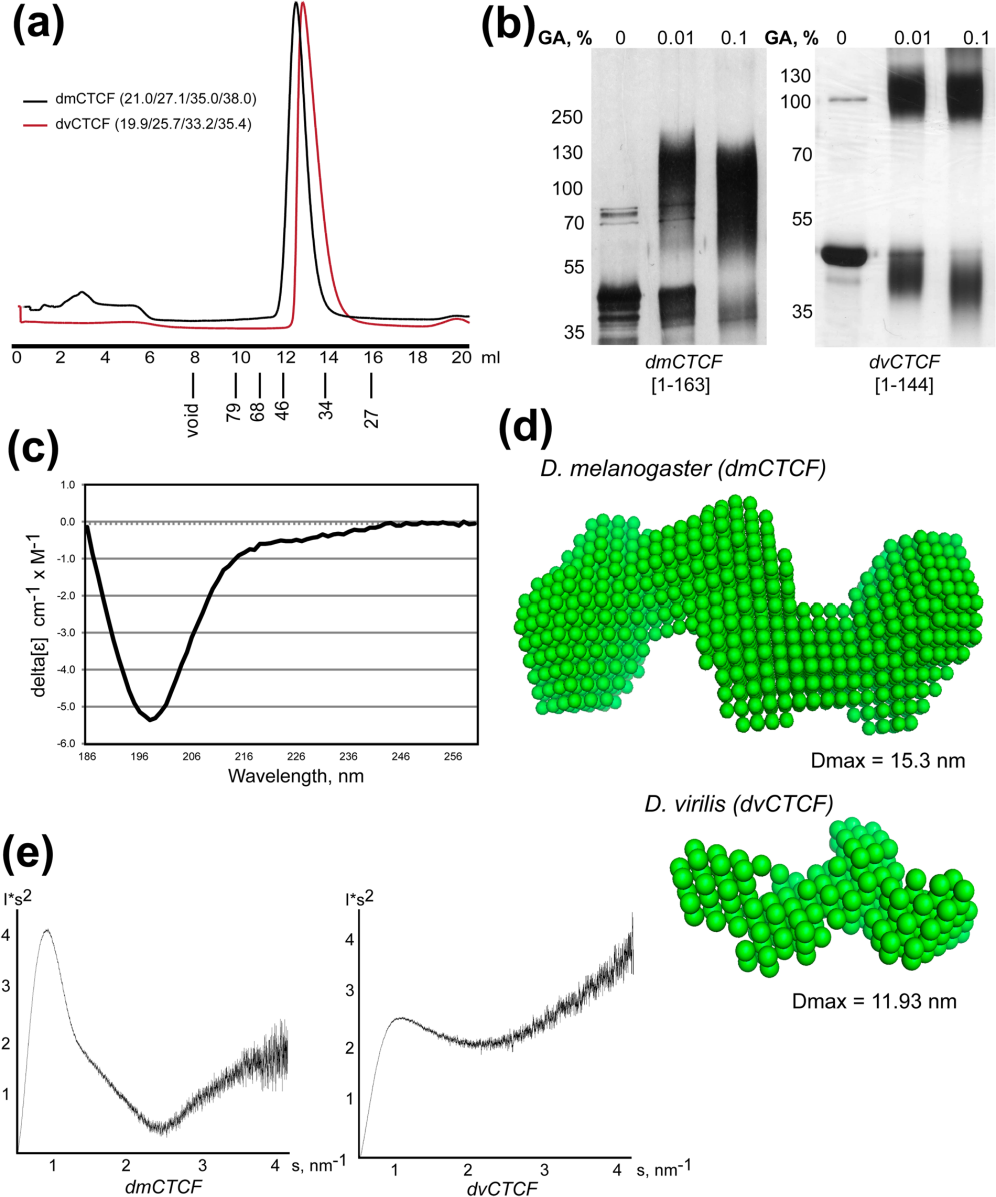
Biochemical and biophysical studies of dCTCF N-terminal domain. (**a**) Superdex S200 size-exclusion chromatography of *Drosophila* CTCF NTDs (without Thioredoxin). SEC measures Stokes radius (Rs) of particles in solution, which depends on the size of particles and their shape. Estimated Rs are calculated for globular and completely unfolded particles, which appear much heavier than could be expected for globular molecules with the same molecular weight^66^. Calculated Stokes radii (Rs, Å) for *Drosophila* CTCF NTDs are shown in brackets (globular monomer/globular dimer/globular tetramer/unfolded monomer). SEC showed that both NTDs have larger Rs than calculated for monomeric and even dimeric globular protein of that molecular weight. Elution volumes of proteins with known Rs are shown under histogram. (**b**) Cross-linking of Thioredoxin-tagged *Drosophila* CTCF NTDs using increasing concentrations of glutaraldehyde (GA). (**c**) The far-UV circular-dichroism spectra of *D. melanogaster* NTD at 20 °C reveals important characteristics of its possible secondary structure. The shape of obtained spectra shows the random coil conformation of *D. melanogaster* NTD. (**d**) *Ab initio* bead model of *D. melanogaster* CTCF N-terminal domain (1–163) (calculated from data obtained at 7.5 mg/ml) and *D. virilis* CTCF N-terminal domain (1–144) (calculated from data obtained at 5.0 mg/ml) obtained by DAMMIN shape reconstruction program based on SAXS data. SAXS provides precise information about the size of macromolecules in solution that is almost independent of their shape. Dmax—maximum dimension of the particles. (**e**) Kratky plot (I*s2 vs s) of SAXS data derived for *Drosophila* CTCF NTDs to assess the folding state of protein molecules^75^. Logarithmic curve suggests unfolded protein, whereas the bell-shaped curve indicates the presence of globular structure. Both Drosophila NTDs demonstrate such bell-shaped appearance (more obvious in case of dmCTCF-NTD), which strongly suggests that these proteins are folded at least partially.

The N-terminal domains (NTDs) from dvCTCF (1–144 aa, 16 kDa) and dmCTCF (1–163 aa, 18 kDa) were expressed in bacteria and tested for dimerization using size-exclusion chromatography (SEC) (Fig. 1a) and cross-linking experiment (Fig. 1b). SEC showed that both NTDs have larger size than calculated for monomeric and even dimeric globular protein of that molecular weight (Fig. 1a). As was shown for dmCTCF NTD^37^, the cross-linking with glutaraldehyde shows that dvCTCF NTD forms dimers (Fig. 1b). Because the values obtained in SEC still are larger than those calculated for dimeric NTDs, they can either form higher-order assemblies that somehow do not cross-link, probably because of the lack of neighboring lysines, or they have unfolded regions that contribute to an increase of the size and shape of the molecule. To study secondary structure, we obtained circular-dichroism (CD) spectrum for dmCTCF-NTD, which revealed a lack of alpha-helices and beta-sheets (Fig. 1c). This observation agrees with secondary structure prediction algorithms that evaluate Drosophila CTCF NTDs as disordered protein domains. It is much more likely they are partially unfolded, therefore resulting in the heavier appearance of these polypeptides in SEC. Interestingly, dimer formation presumes the existence of a stable fold, which these polypeptides should adopt without typical secondary structure elements.

To further assess the oligomeric state and check the monodispersity of the purified dmCTCF NTD sample, we used Dynamic Light Scattering (DLS). The size distribution of both samples contained only one narrow peak. Estimated hydrodynamic radius (Rh) value varies in range 4.4–4.6 nm and corresponds to molecular weights of 110 kDa. However, DLS calculations of molecular weight as well as SEC are highly sensitive to the shape of the molecule, which often leads to the overestimation of protein molecular weight, so the value of 110 kDa corresponding to hexamer could result from multimerization as well as from the presence of unfolded regions.

To determine the correct oligomerization status of NTDs, we used a Small-Angle X-ray Scattering (SAXS) approach. Calculated molecular weights of dmCTCF NTD were in the range of (71–83) kDa corresponding to tetramer (monomer Mw is 18 kDa) in agreement with SEC data rather than with cross-linking experiments (Table 1). For dvCTCF NTD, the calculated molecular weight was in the range of 29–35 kDa, corresponding to dimer (monomer Mw is 16 kDa). Several possible low-resolution models were built based on scattering profiles (Fig. 1d). Two-fold symmetry of the model suggests that tetramer is assembled from two dimers consisting of tightly bound monomers that effectively cross-link to each other. The elongated shape of the tetramer explains a heavier molecular weight of 100 kDa, roughly calculated from the SEC profile (Fig. 1d). Model of dimeric dvNTD has a smaller volume, in accordance with a lower molecular weight of assemblies, and roughly resembles the dimeric part of the dmCTCF NTD (Fig. 1d). Kratky plot of the SAXS profile shows that both Drosophila NTDs are folded at least partially (Fig. 1e). Because the CD experiment does not show any secondary structure elements in dmCTCF-NTD, it seems likely that both NTDs have unusual spatial fold lacking secondary structure elements. From the overall shape of the Kratky plot, we can conclude that *Drosophila* CTCF NTDs has overall globular structure formed by unfolded regions, which explains why the SEC profile is heavier than could be expected for globular dmNTD tetramer or dvNTD dimer.

**Table 1:** Molecular weight of protein particles calculated from SAXS data using extrapolated I_0_ scattering intensity and protein standards of known Mw as described^71^.

CTCF-NTD	MW of the monomer, kDa	Estimated MW in solution, kDa
*dmCTCF*	18.7	71.0–83.0
*dvCTCF*	16.3	29.0–35.0
*dpCTCF*	25.1	33.0–41.0
*amCTCF*	26.1	25.0–29.0
*cgCTCF*	19.9	27.5–33.5
*spCTCF*	35.5	49.0–54.0
*skCTCF*	44.2	52.0–62.0
*ciCTCF*	31.6	37.0–47.0
*hsCTCF*	31.4	45.0–51.0

Scattering parameters for the N-terminal domains of CTCF from various species are shown in Table S1.

To provide further insight into structural features underlying their multimerization we studied CTCF NTDs from two *Drosophila* species (*D. virilis* and *D. melanogaster*) using 2D NMR spectroscopy. The ^15^N,^1^H HSQC spectra for ^15^N-labelled dmCTCF NTD and dvCTCF NTD were found to have similar features for both proteins (Fig. S3). The spectra undoubtedly indicate some signals typical for folded proteins. These signals exhibit significant line broadening, which is not fully eliminated by increasing the temperature to 50 °C (Fig. S4). Even at this temperature, there are significant differences in the signal line widths of the residues located in structured and unstructured parts. Such behavior is typical for large structured proteins due to their slow tumbling. Putting it all together we can conclude that both dmCTCF and dvCTCF NTDs have a similar structural organization with a structured protein core, but at least 2/3 of the protein chains represent an unstructured coil.

### N-terminal domains of CTCF show unstructured nature in all tested organisms from all major phylogenetic groups of higher metazoans

**Figure 2:**
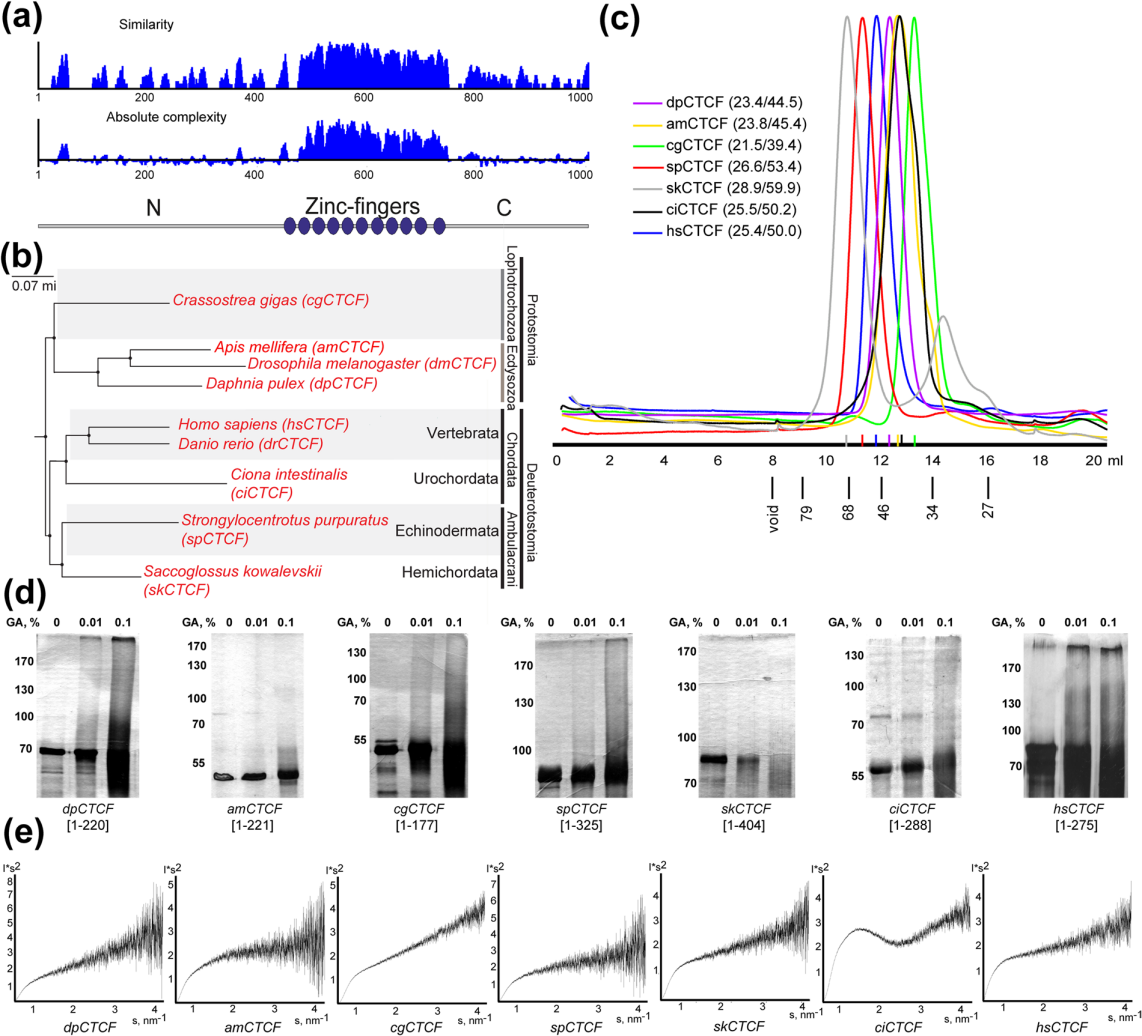
(**a**) Summary of multiple sequence alignment of CTCF proteins used in this study. The similarity is a score of how similar each amino acid or groups of amino acids are across the whole alignment. The absolute complexity is the average of the pairwise alignment scores using the substitution matrix chosen in the alignment setup. Overall domain structure of CTCF proteins is shown below. (**b**) Positions of species selected for this study on the phylogenetic tree of metazoans (adapted from^38^). (**c**) Superdex S200 size-exclusion chromatography of CTCF NTDs (without Thioredoxin). Elution volumes of proteins with known Rs values (Å) are shown. Calculated Rs values for NTDs are shown in brackets (globular/unfolded monomer). (**d**) Cross-linking of Thioredoxin-tagged NTDs using increasing concentrations of glutaraldehyde (GA). (**e**) Kratky plot (I*s2 vs s) of SAXS data derived for CTCF NTDs. The bell-shaped curve suggests that polypeptide is folded, whereas the logarithmic shape is a sign of random coil conformation.

Since the cluster of zinc-finger domains of CTCF proteins is the only domain (Fig. 2a) that exhibits the high level of conservation within higher metazoans^17^, we asked whether their NTDs could display multimerization activity like *Drosophila* NTDs despite a lack of evolutionary conservation. To answer this question, we cloned the NTDs of CTCFs from well-characterized representatives with the known genomes of diverse phylogenetic groups of higher metazoans (Fig. 2b). The functional role of CTCF in most of these groups has not been characterized yet. *D. pulex* (dpCTCF, water flea) and *A. mellifera* (amCTCF, European honey bee) are Arthropods, both belonging to Ecdysozoa phylum of Protostomia. *C. gigas* (cgCTCF) belongs to mollusks, which also are Protostomes, together with annelids comprising the Lophotrochozoa phylum (Fig. 2b). A Deuterostomia superphylum is comprised of three phyla — Chordata, Hemichordata and Echinodermata (Fig. 2b). *S. purpuratus* (spCTCF) is a representative of Echinodermata. The role of spCTCF in the establishment of TAD borders was shown earlier^21^. *S. kowalewski* (skCTCF) is the marine invertebrate, a representative of Hemichordata, being close to basal Chordates. This organism also displays signs of reduction^38^. *C. intestinalis* is lower Chordata. *C. intestinalis* (ciCTCF) genome was sequenced in 2002 and despite being about 1/20 of the human genome by size, it contains an almost complete set of genes found in vertebrates, although many organs were reduced or secondary lost^39^. In vertebrates, CTCF proteins are described as key organisers of chromosomal architecture. Consistent with the important role in transcription regulation, vertebrata CTCFs have high homology in all characterized representatives (Fig. S2). We cloned the NTDs of CTCFs from human (hsCTCF) and zebrafish (drCTCF), which display the maximum difference in amino acid sequences between vertebrates. Despite sequence differences, human CTCF was recently found to be able to rescue zebrafish CTCF knockout, which otherwise is lethal^40^. We did not find CTCF homologs in Radiata (Cnidaria and Ctenophora), basal metazoans — Porifera and Placozoa. Emergence of CTCF protein is often associated with origin of Bilaterian metazoans, but CTCF homologs were not found in flatworms, presumably due to the secondary loss. Also, CTCF is absent in several clades of nematodes^29^.

Bioinformatic analysis of selected domains using a PredictProtein algorithm^41^ revealed that all of them are predicted to be mostly disordered. For subsequent biochemical and biophysical analysis, CTCF NTDs were expressed in E.coli. Unfortunately, we were unable to express in bacteria a sufficient amount of drCTCF NTD. Values measured by SEC for all proteins appeared larger than could be expected for monomeric globular form and close to expected for unfolded proteins (Fig. 2c). Chemical cross-linking revealed no multimer formation (Fig. 2d), suggesting that domains are possibly intrinsically disordered, in agreement with previous studies of N-terminal domain from human CTCF protein^30^.

The SAXS technique was applied to provide further information about the structure of CTCF NTDs. We summarize the results of SAXS data analyses in Table 1. Despite the lack of stable fold in solution and absence of multimers revealed by cross-linking experiments, several proteins demonstrate heavier estimated weight in SAXS experiments. Analysis of SAXS data from spCTCF and hsCTCF NTDs suggests possible aggregation of molecules; however, these assemblies have stable size. At the same time, molecular weight calculated from SAXS data is only about 1.5 times higher than expected from the amino acid sequence, which can be explained by the fact that molecules are intrinsically disordered. It has been shown that human CTCF NTD is monomeric in solution^30^. Our cross-linking experiments also did not reveal high-molecular weight product (possibly because of the lack of neighbouring lysines), but SAXS data (reproduced in two measurements of independent protein preparations) suggest that assemblies with a larger volume can form under several conditions. Chemical cross-linking with glutaraldehyde and EGS along with size-exclusion chromatography were used to test possible change in oligomerization status of hsCTCF NTD induced by concentration to 10 mg/ml and freeze-thaw cycles, but still, we did not observe any detectable presence of hsCTCF-NTD multimers. SAXS is extremely sensitive to the presence of high-molecular-weight particles, so most likely, these observations could be attributed to small amounts of aggregates in samples. NTDs of *D. pulex* and *S. purpuratus* also have slightly larger molecular weight than calculated for monomer, but both are unstructured (as can be seen from the Kratky plot (Fig. 2e)). For all NTDs, Dmax (maximum linear size of particles) was several times higher than Rg (averaged distance to all atoms from the center of mass of the molecule), suggesting the elongated shape of particles. Analysis of SAXS data using the Kratky plot (Fig. 2e) revealed a bell-shaped curve only for *Ciona* and *Drosophila* (Fig. 1e) NTDs, showing that these polypeptides are at least partially folded, but other proteins had the logarithmic shape of the plot that is rather appropriate for disordered protein chains, which explains their heavier appearance on SEC profile.

Thus, *Drosophila* CTCF NTDs have the unique ability to form multimers *in vitro* among metazoans, even in contrast to related *Apis mellifera*. They adopt an unusual fold with the absence of secondary structure elements.

### Testing dimerization of the N-terminal domains of CTCFs in heterologous *in vivo* systems

Lack of the homodimerization ability of CTCF NTDs in experiments *in vitro* does not exclude this property of NTDs *in vivo*. To test this possibility, we used two different approaches. The first was a yeast two-hybrid assay (Y2H). Sequences encoding the NTDs were fused in-frame to the yeast GAL4 DNA-binding domain (BD) and activation domain (AD). Because steric hindrance can interfere with transcriptional activation in the two-hybrid system, the NTD sequences were placed at both the N-terminus (NTD-AD and NTD-BD) and the C-terminus (AD-NTD and BD-NTD) of the fusion protein.

**Figure 3:**
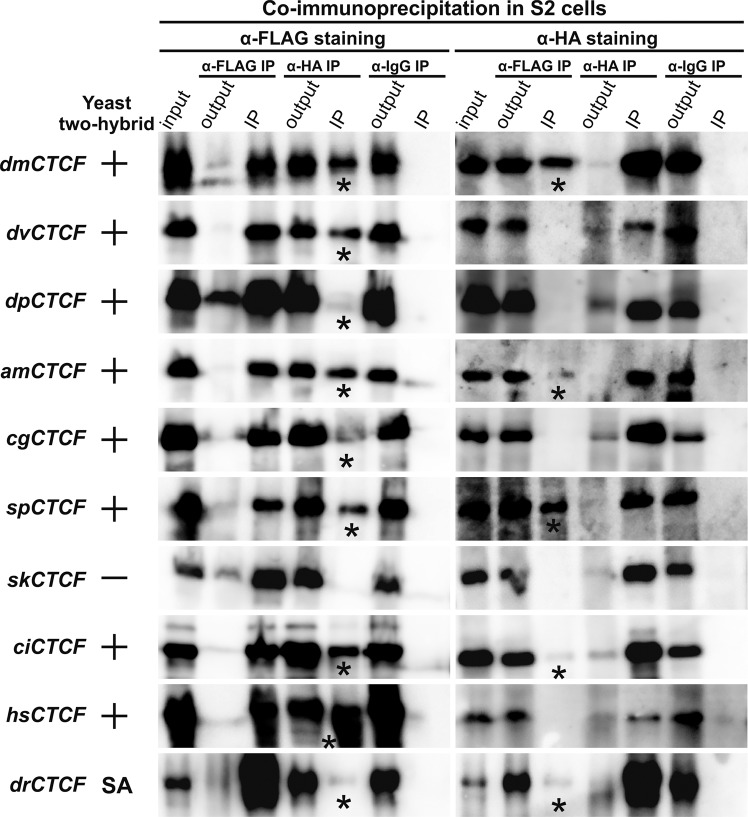
Analysis of CTCF proteins N-terminal dimerization *in vivo*. Summarized results for NTDs CTCF interactions in the yeast two-hybrid assay are presented at the left. NTD CTCFs were fused to the N- and C-termini of the GAL4 DNA-binding and activating domains. The resulted plasmids were tested for interaction. The results of Y2H analysis are shown in columns. The “+” and “−” signs indicate an interaction or the absence of interaction, respectively. The GAL4 activating and GAL4 DNA-binding domains alone were used as negative controls. SA – self-activation variant of NTD CTCF. Co-immunoprecipitations of NTDs CTCF fused with 3 × FLAG or 3 × HA were performed in S2 cells. *Drosophila* S2 cells were cotransfected with 3 × FLAG- and 3 × HA-fused plasmids. Antibodies against 3 × FLAG or 3 × HA were used for immunoprecipitation of protein extracts (IP). Nonspecific IgG antibodies were used as a negative control. The presence of HA- or FLAG-tagged proteins were studied by Western blotting. ‘Input’ refers to samples of the initial protein extract; ‘output’ refers to the supernatant after the removal of immunoprecipitate (IP). Specific IP signal with partner protein is indicated with an asterisk. Detailed results are presented in Table S2.

For dmNTD, we had previously found that a positive result was observed in only one configuration, dmNTD-BD and AD-dmNTD (Bonchuk *et al*.^37^). Here we confirmed this observation for the dm-NTD and found that other NTDs also were able to interact only in one of four tested configurations (Table S2). The most of the tested NTDs (am, cg, sp, ci, dp and h) demonstrated the pairing ability (Fig. 3). We did not observe the interaction between the skNTDs. We also failed to test dimerization of drNTD due to strong self-activation induced by the NTD sequence fused with the BD.

To confirm the Y2H results by independent assay, we analysed co-immunoprecipitation of 3 × FLAG and 3 × HA-tagged NTDs in transfected S2 cells (Fig. 3). Each NTD was fused with either 3 × FLAG or 3 × HA epitope and co-transfected into S2 cells. After immunoprecipitation with HA-Sepharose, the bands corresponding to homodimers of NTDs were detectable for all NTDs (strong signal for dm, dv, am, sp, ci, hs and weak for dp, cg, dr), the exception being skCTCF. At the same time, in the reverse experiment with FLAG-Sepharose, we observed homodimer bands only for part of NTDs (strong signal for dm, sp and weak for am, ci, dr). Such an unstable result can be explained by some steric difficulties in immunoprecipitation of proteins. Taken together, the results of Y2H and co-immunoprecipitaion assay show that the NTDs of CTCFs from different organisms are capable of homodimerization. Only skNTD did not show the ability to form dimers in both used approaches.

## Discussion

The CTCF belongs to transcription factors with an arranged array of C2H2 domains. In contrast to TFs of other classes, C2H2 proteins typically bind to 12–20 bp sequences^42–44^. The C2H2 domains of CTCF are most conserved among this class of the proteins, suggesting the model that CTCF is the ancestral protein from which other C2H2 proteins originated during evolution. According to a hypothesis, CTCF appeared in evolution when long-distance interactions between regulatory elements had emerged in transcription regulation^21,45^. It seems likely that many other C2H2 proteins originating from CTCF are also involved in the organization of chromosomal architecture. Some of these proteins were discovered in *Drosophila* initially as insulator proteins Su(Hw), Zw5, Pita and Zipic^46–48^.

Many C2H2 proteins have N-terminal homodimerization domains. In arthropods and vertebrates^49–51^, expansion of different domains was observed: ZAD and SCAN, respectively, which exhibit the ability to predominantly form homodimers. It was demonstrated that homodimerization ZAD from three C2H2 proteins (Pita, ZIPIC and Zw5) determines the specificity of long-range interactions^52^. C2H2 proteins can also have other types of multimerization domains. For example, the C2H2 protein Opbp has the N-terminal C2H2 domain that can form homodimers and is involved in distance interactions^53^. It was recently shown that YY1 participates in enhancer-promoter interactions by forming oligomers^54^. Interestingly, YY1 contains 3 C2H2 domains at the C-terminus that are involved at the same time in oligomerization and DNA binding^55^. Another protein, LDB1, the Lim domain binding 1 protein, contains a dimerization domain that plays an important role in enhancer-promoter interactions in various developmental pathways^56–58^.

Here, we found that non-conserved N-terminal domains of CTCFs in all tested metazoan are intrinsically unstructured *in vitro*, but in most cases, they show potency to self-association *in vivo*. Only in the case of CTCF isolated from acorn worm (*Saccoglossus kowalewski*) did we not observe homodimerization of N-terminal domain *in vivo*. Thus, most N-terminal CTCF domains keep structural and functional properties despite the non-conservation of sequences during evolution. Exceptions are CTCFs from *Drosophila melanogaster* and *virilis* (Drosophilids). Those N-terminal domains are folded *in vitro* despite the lack of secondary structure elements. It seems likely that such domain organization was adopted in Drosophilids, as the N-terminal domain of CTCF in honey bee is intrinsically disordered. Even in Drosophilids, the structure of N-terminal domains varies between tested species: N-terminal domain of *Drosophila melanogaster* forms tetramer, but N-terminal domain of *Drosophila virilis* forms only dimer.

The crucial role of CTCF in supporting specific distance interaction in mammalians might suggest the ability of CTCF to homodimerize. It was shown that hCTCF can dimerize by purification of FLAG-HA-tagged CTCF complex and in the yeast two-hybrid assay^59^. However, attempts to find the dimerization domain in hCTCF that can support specific distance interactions have thus far been unsuccessful. It was shown in pulldown experiments that the C-terminal part of one CTCF binds to the C2H2 zinc-finger domains of another CTCF^60^, but the specificity of this interaction has not been proven. It was also found that some RNAs can interact with 10 and 11 ZF and induce oligomerization of the CTCF protein^61^. Because many C2H2 domains can with relatively low specificity interact with RNAs^62–64^, the involvement of RNAs in protein multimerization does not explain how CTCF can support specific distance interactions.

Unstructured N-terminal regions of CTCFs are a good candidate for the role of a domain that supports specific distance interactions between CTCF sites. The strength of pairing between unfolded NTDs can be easily regulated by various post-translational modifications of amino acid residues, which are crucial for effective stimulation/repression of enhancer-promoter interactions. The NTDs in CTCFs lack secondary structure and sequence similarity, therefore, making it impossible to identify such domains using bioinformatics approaches. Thus, there is a probability that unstructured domains are widely distributed at the N-terminal ends of C2H2 proteins, which, however, can only be verified experimentally. Further studies are required to understand the role of the N-terminal domains in the organization and regulation of distance interactions mediated by CTCFs.

## Materials and Methods

### Plasmid construction

CTCF homologues were identified using BLAST search by similarity with zinc-finger domain of *Drosophila* and human CTCF proteins. For protein purification purposes, protein fragments were PCR-amplified using corresponding primers (see Table S3) and subcloned into modified pET32a(+) vector (Merck Biosciences) in-frame with TEV-cleavable Thioredoxin-6xHis-tag. Adult bees (*Apis mellifera*) were obtained from a local apiary, oysters (*Crassostrea gigas*) were purchased at a local food store, and *Daphnia pulex* culture was purchased at a pet shop. RNA was isolated using TRIzol reagent, and cDNA was obtained with reverse transcription with oligo(dT) primer following standard protocols. For other cDNAs sources, see the acknowledgements.

### Protein expression and purification, size-exclusion chromatography and chemical cross-linking

Protein expression and purification were performed using standard procedures, as described previously^37^. Stable isotope-labelled proteins were expressed according to^65^ and purified using the same procedure as for native proteins. Size-exclusion chromatography was performed as described^37^ using Superdex 200 10/300GL columns (GE Healthcare). Expected Rs values for globular and unfolded proteins were calculated as described^66^. Chemical cross-linking of proteins was carried out with glutaraldehyde as described previously^37^.

### Circular dichroism

Circular-dichroism measurements were performed using Chirascan instrument (Applied Photophysics, UK). The instrument was calibrated using Camphor-10-sulfonic acid, according to^67^. Measurements were made in 0.1 cm isolated cuvette at sample concentration of 0.05 mg/ml at 20 °C. Sample concentration was calculated from peptide-bond extinction values at 205, 206, 210 и 215 nm^68^.

### SAXS measurements and data processing

Synchrotron radiation X-ray scattering data were collected using standard procedures on the BM29 BioSAXS beamline at the ESRF (Grenoble, France) at a wavelength of 0.099 nm. The 2D detector Pilatus1M and sample to detector distance 2.87 m were used to acquire scattering data within the momentum transfer (s) covering a range of 0.033–4.9 nm-1 (s = 4πSinθ/λ where 2θ is the scattering angle). Data collection and processing were performed in an automated manner using dedicated beamline software BsxCuBE. The samples were measured at least at two concentrations. A volume of 30 μl of sample solution was placed in a 1.8-mm-diameter quartz capillary with a few tens of microns wall thickness. Thirty consecutive frames with 1 s exposure were collected from the sample at constant temperature 277 K without observing any radiation damage (characterized by systematic deviations in consecutive scattering curves). Solvent scattering was measured to allow for subtraction of the background scattering. The data from consecutive frames were inspected, normalized to the incident beam intensity and averaged in PRIMUS^69^. Data processing and analysis were done with the ATSAS program suite for small angle scattering from biological molecules^70^. The subtraction of the buffer scattering was done manually by program subtrNc. The radius of gyration Rg of protein molecule in solution was evaluated using the Guinier approximation at small angles (s < 1.3/Rg), assuming the intensity I(s) to be equal to I_0_ *exp*(−(sRg)^2^/3). To evaluate the maximum particle dimension Dmax, the pair-distance distribution function P(r) was generated with the program GNOM so that the Rg value of protein samples had to agree with that determined from the Guinier region in PRIMUS. The molecular mass (MM) of the protein was calculated using extrapolated I0 scattering intensity and protein standards of known Mw as described^71^. Low-resolution *ab initio* structure models of CTCF(1–163aa) protein representing the protein as an ensemble of dummy atoms were constructed by program DAMMIN. The program was used to build a compact configuration of beads inside a sphere of Dmax diameter with χ = 1.05 minimal discrepancy between intensity of experimental data and that calculated from generated model.

### Dynamic light scattering (DLS)

Dynamic light scattering (DLS) measurements were performed using an instrumentation of Dyna Pro Titan (Wyatt Technology Corporation). Light scattering analysis was performed using a laser wavelength of 832 nm, quartz cuvette of 20 µl volume, temperature controlled DynaPro instrument at 4 °C and Dynamics software. The protein samples were analysed in 20 mM TrisHCl buffer (pH 7.4), 200 mM NaCl, containing 1 mM β-mercaptoethanol and 10% (w/v) glycerol. The protein was concentrated up to 1 and 7 mg/ml and filtered prior to the measurements. Sequences of 10 sample acquisitions with 1 s time duration were collected at each concentration. The value of the solution viscosity was chosen out from the corresponding table of the instrument. The hydrodynamic radius (Rh) was evaluated by Stokes-Einstein equation from the autocorrelation function of DLS measurements following standard procedures. The average MM was estimated using default Mark-Houwink parameters for a hard sphere.

### NMR spectroscopy

The NMR samples in concentrations of 0.2–0.5 mM for ^15^N-labelled dmCTCF and dvCTCF were prepared in 95% H_2_O/5% D_2_O, 20 mM NaCl, 20 mM sodium phosphate buffer (pH 7.0 or 6.5), and 0.02% NaN_3_. All spectra were recorded on Bruker AVANCE 600 MHz spectrometer (Moscow State University). For 2D NMR the SOFAST HMQC pulse program was used^72^. The acquired data were processed using NMRPipe^73^, and analyzed using NMRFAM-Sparky software^74^.

### Yeast two-hybrid assay

Yeast two-hybrid assay was carried out using yeast strain pJ69-4A (MATa trp1-901 leu2-3,112 ura3-52 his3-200 gal4Δ gal80Δ GAL2-ADE2 LYS2::GAL1-HIS3 met2::GAL7-lacZ), as described previously^52^.

### Co-immunoprecipitation assay

Protein extracts were prepared from S2 cells cotransfected by 3 × FLAG- and 3 × HA-fused plasmids with MACSfectin (Miltenyi Biotec). Coimmunoprecipitaion assay was described previously^52^.

## Supplementary information

Supplementary data.

## Data Availability

All data generated or analysed during this study are included in this published article and its Supplementary Information files.
