# Supplementary material for: N-terminal domain of the architectural protein CTCF has similar structural organization and ability to self-association in bilaterian organisms
Source: Sci Rep. 2020 Feb 14;10:2677. doi: 10.1038/s41598-020-59459-5 (PMC7021899; doi:10.1038/s41598-020-59459-5)
Supplement: Supplementary file 1 — Supplementary data. [file 41598_2020_59459_MOESM1_ESM.pdf]

N-terminal domain of the architectural protein CTCF has similar structural organization and ability to self-association in bilaterian organisms

Artem Bonchuk, Sofia Kamalyan, Sofia Mariasina, Konstantin M. Boyko, Vladimir O. Popov, Oksana Maksimenko, Pavel Georgiev

SUPPLEMENTARY INFORMATION

TABLES

Table S1. Scattering parameters for the N-terminal domains of CTCF from various species. Rg is radius of gyration (average of square center-of-mass distances in the molecule), Dmax – maximum dimension of the particles, Vp – Porod volume – volume of the particles.

Polypeptide	Sample concentration, mg/ml	Rg, nm	Dmax, nm	Vp, nm3	Estimated molecular weight in solution, kDa	Molecular weight of the monomer, kDa
Drosophila melanogaster [1-163]	1	4.65	15.2	110.5	71.0-83.0	18.7
	7.5	5	16	125.5	67.0-77.0	
Drosophila virilis [1-144]	1	2.7	9.4	47.6	29.0-35.0	16.3
	5	3.6	11.93	96.2	31.0-37.0	
Daphnia pulex [1-220]	1	4.8	18.5	69.8	33.0-41.0	25.1
	9	4.6	15.84	58.4	27.0-33.0	
Apis mellifera [1-221]	1	3.6	10.94	50.1	25.0-29.0	26.1
	4	4	13.7	39.6	27.0-33.0	
Crassostrea gigas [1-177]	1.35	4.36	14.94	42.85	27.5-33.5	19.9
	5.4	4.18	14.63	41.8	25.0-31.0	
Strongylocentrotus purpuratus [1-325]	1	5.9	20.9	49.9	49.0-54.0	35.5
	10	5.2	18.2	40.5	27.0-31.0	
Saccoglossus kowalevski [1-404]	1	5.2	16.0	99.2	52.0-62.0	44.2
	5.5	5.8	23.1	119.6	36.0-44.0	
Ciona intestinalis [1-288]	1.65	4.3	15.04	61.73	37.0-47.0	31.6
	6.5	3.83	13.43	45.9	31.0-37.0	
Homo sapiens [1-264]	1	5.4	28.44	301.11	45.0-51.0	31.4
	5	4.9	28.2	305.22	46.0-52.0	

Table S2. Detailed results of yeast two-hybrid and co-immunoprecipitation analysis for each NTD CTCF pair.

Relative N- or C- terminal position of AD/BD is shown. AD stands for GAL4 activation domain, BD—GAL4 DNA-binding domain, 3AT—3-amino-triazol, competitive inhibitor of reporter HIS3 gene product. Grey cells mark self-activation. In these cases, additional 3AT growth (2, 5, 10, 15, 20 mM) assay was carried out. Immunoblots demonstrate full-sized and additional co-immunoprecipitation repeats for some NTD CTCF pairs.

dmCTCF	'medium-3'		
	BD	BD-dm	dm-BD
AD	-	-	-
AD-dm	-	-	+
dm-AD	-	-	-

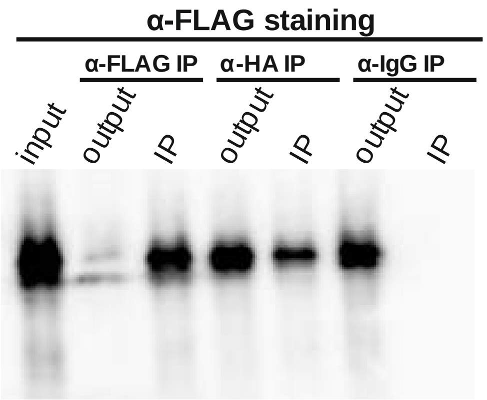

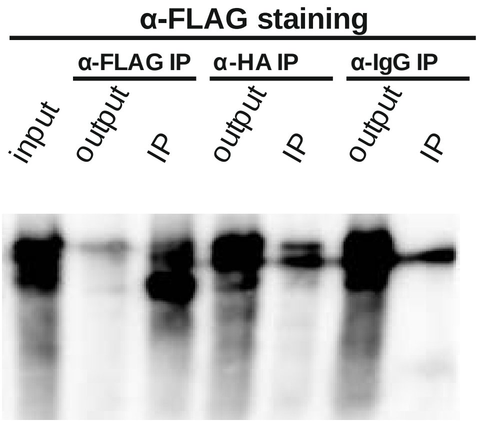

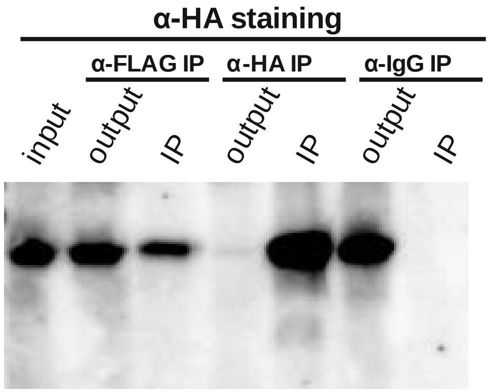

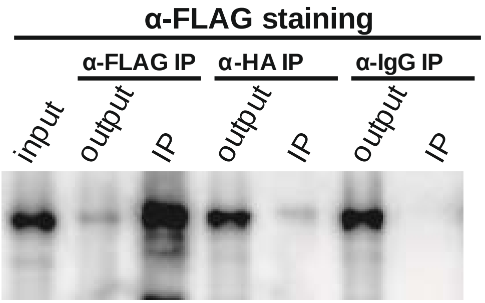

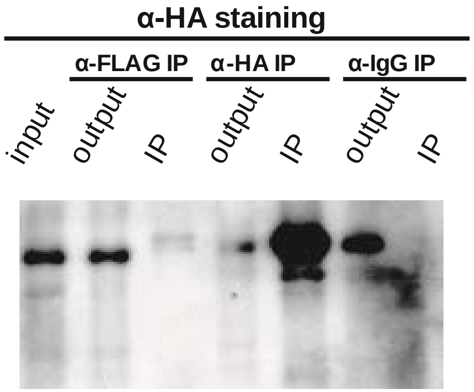

dvCTCF	'medium-3'		
	BD	BD-dv	dv-BD
AD	-	-	-
AD-dv	-	+	-
dv-AD	-	-	-

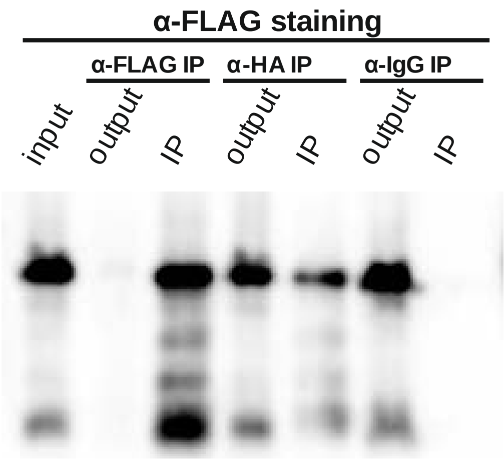

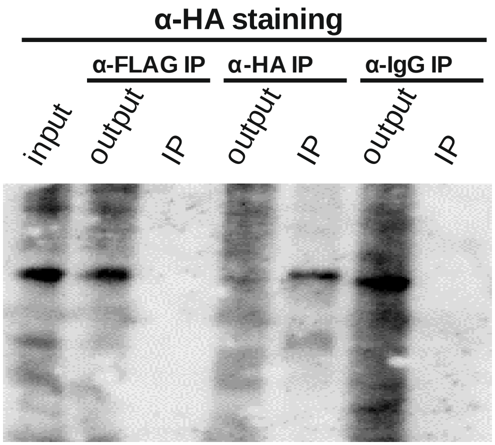

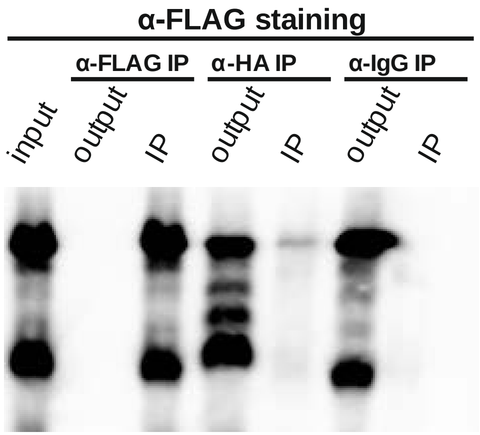

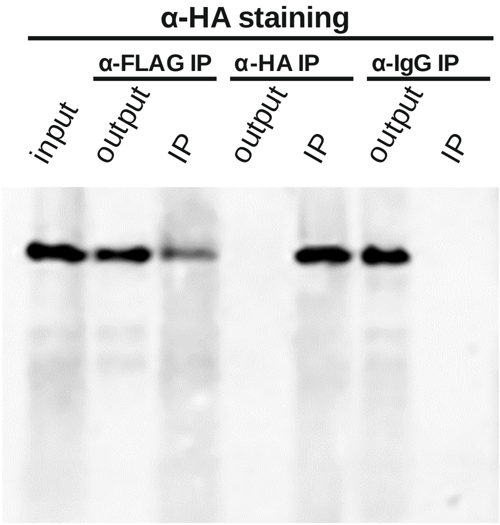

dpCTCF	'medium-3'			'medium-3' + 2mM 3AT			'medium-3' + 20mM 3AT		
	BD	BD-dp	dp-BD	BD	BD-dp	dp-BD	BD	BD-dp	dp-BD
AD	-	+	+	-	+	+	-	-	-
AD-dp	-	+	+	-	+	+	-	-	+
dp-AD	-	+	+	-	+	+	-	-	-

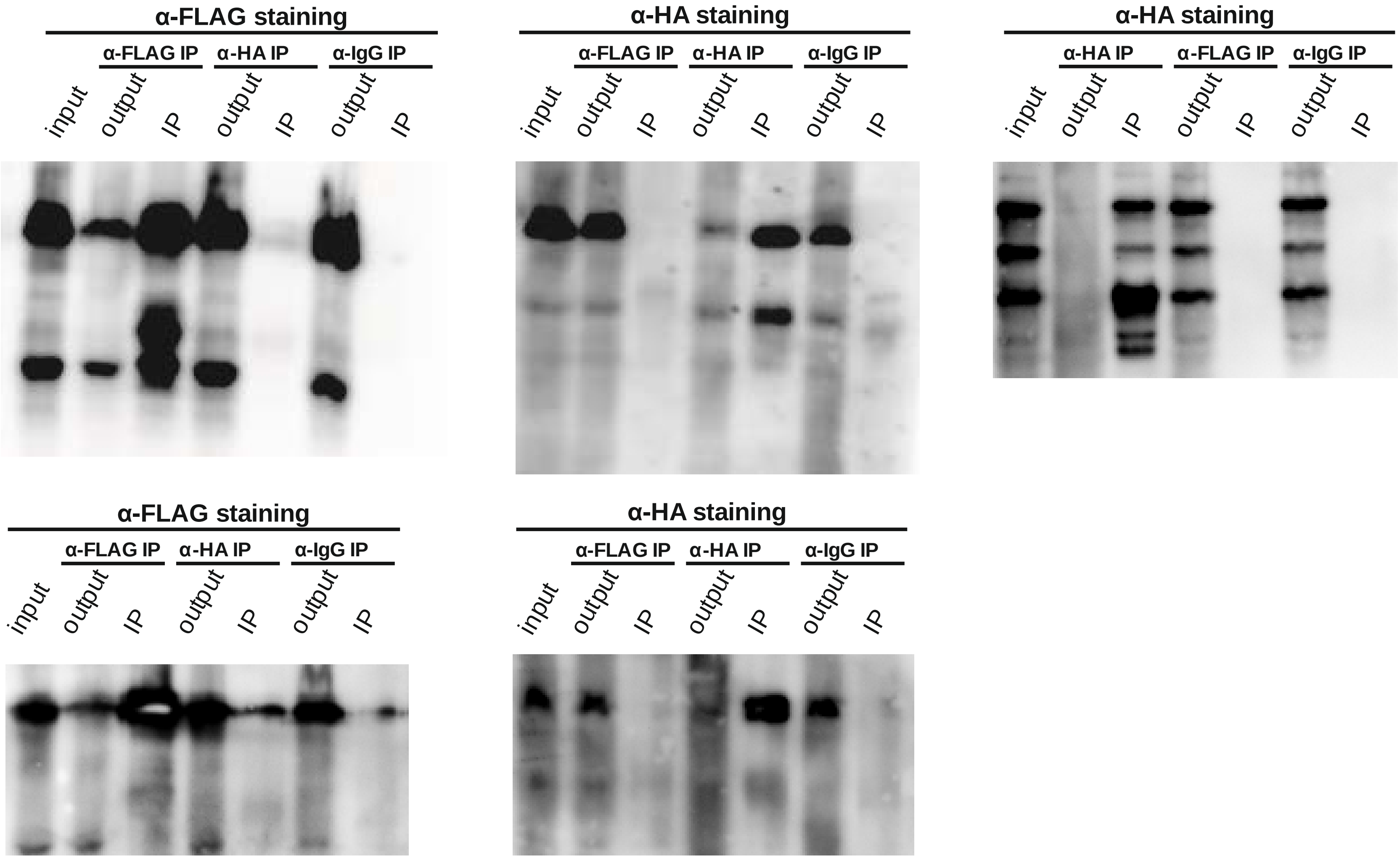

amCTCF	'medium-3'			'medium-3' + 2mM 3AT			'medium-3' + 5mM 3AT			'medium-3' + 10mM 3AT			'medium-3' + 15mM 3AT			'medium-3' + 20mM 3AT		
	BD	BD-am	am-BD	BD	BD-am	am-BD	BD	BD-am	am-BD	BD	BD-am	am-BD	BD	BD-am	am-BD	BD	BD-am	am-BD
AD	-	+	-	-	+	-	-	+	-	-	+	-	-	+	-	-	-	-
AD-am	-	+	-	-	+	-	-	+	-	-	+	-	-	+	-	-	-	-
am-AD	-	+	-	-	+	-	-	+	-	-	+	-	-	+	-	-	+	-

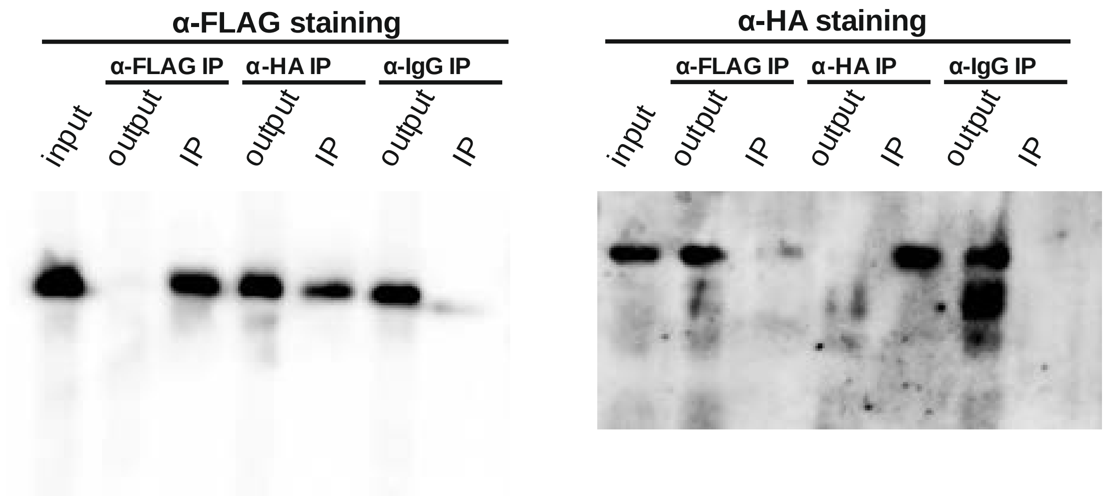

cgCTCF	'medium-3'			'medium-3' + 2mM 3AT			'medium-3' + 5mM 3AT			'medium-3' + 10mM 3AT			'medium-3' + 15mM 3AT		
	BD	BD-cg	cg-BD	BD	BD-cg	cg-BD	BD	BD-cg	cg-BD	BD	BD-cg	cg-BD	BD	BD-cg	cg-BD
AD	-	+	+	-	+	+	-	+	+	-	+	+	-	-	-
AD-cg	-	+	+	-	+	+	-	+	+	-	+	+	-	-	-
cg-AD	-	+	+	-	+	+	-	+	+	-	+	+	-	+	-

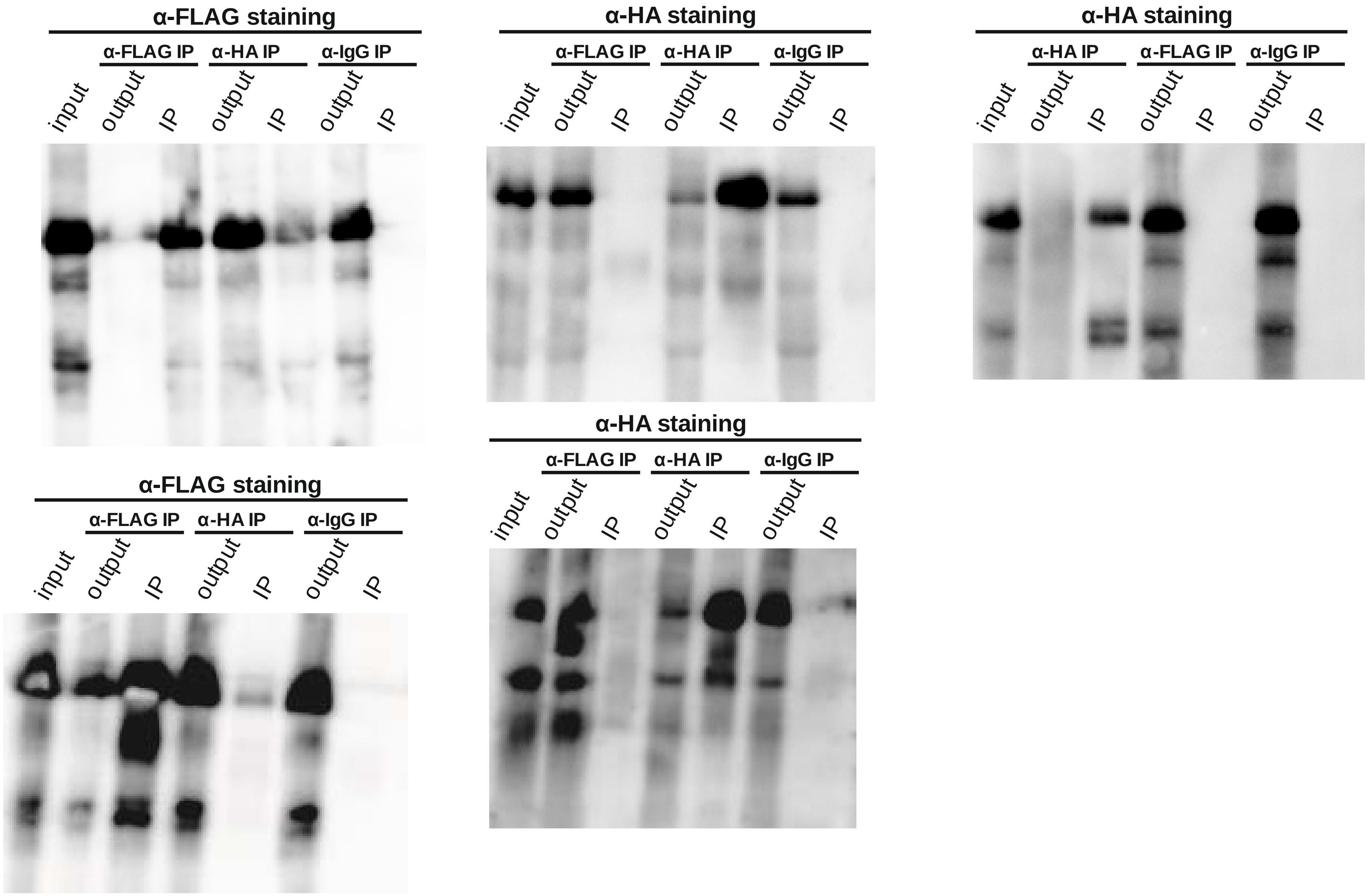

spCTCF	'medium-3'			'medium-3' + 2mM 3AT			'medium-3' + 5mM 3AT			'medium-3' + 10mM 3AT			'medium-3' + 15mM 3AT			'medium-3' + 20mM 3AT		
	BD	BD-sp	sp-BD	BD	BD-sp	sp-BD	BD	BD-sp	sp-BD	BD	BD-sp	sp-BD	BD	BD-sp	sp-BD	BD	BD-sp	sp-BD
AD	-	+	+	-	+	+	-	+	+	-	+	+	-	+	+	-	-	-
AD-sp	-	+	+	-	+	+	-	+	+	-	+	+	-	+	+	-	+	-
sp-AD	-	+	+	-	+	+	-	+	+	-	+	+	-	+	+	-	-	-

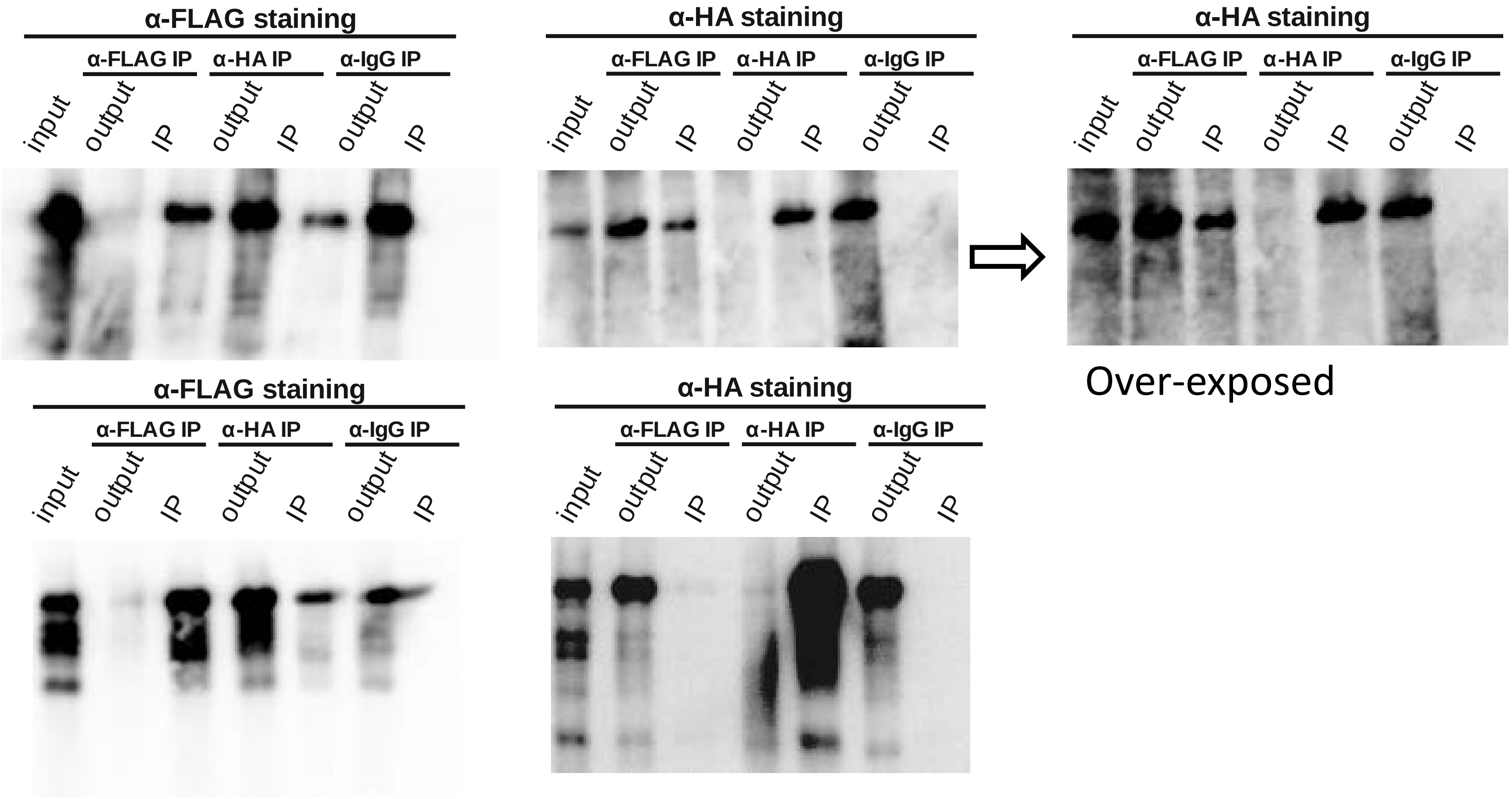

skCTCF	'medium-3'			'medium-3' + 2mM 3AT		
	BD	BD-sk	sk-BD	BD	BD-sk	sk-BD
AD	-	-	-	-	-	-
AD-sk	-	-	-	-	-	-
sk-AD	-	-	-	-	-	-

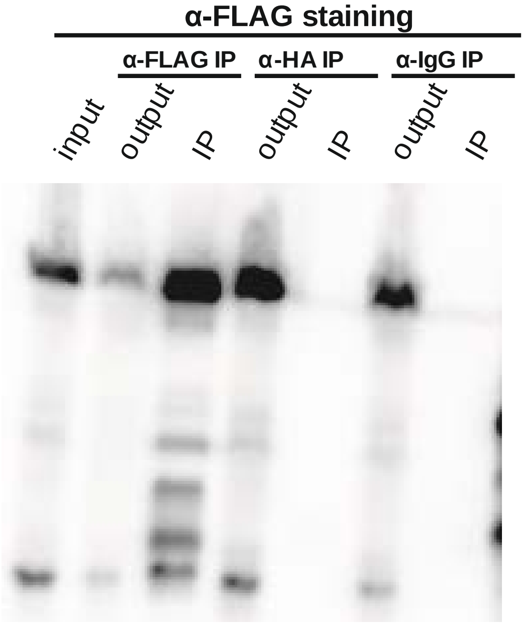

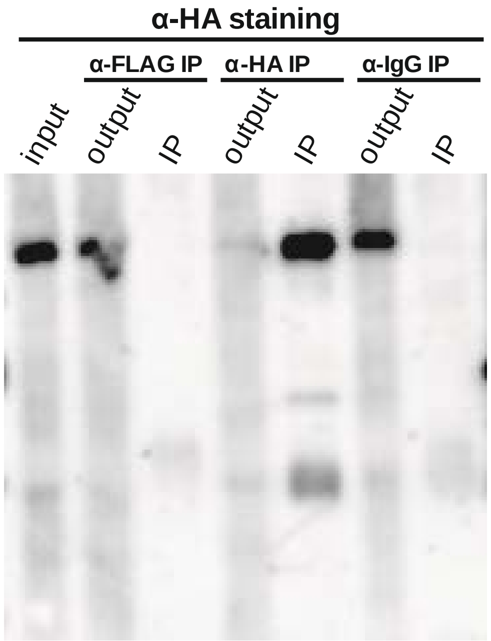

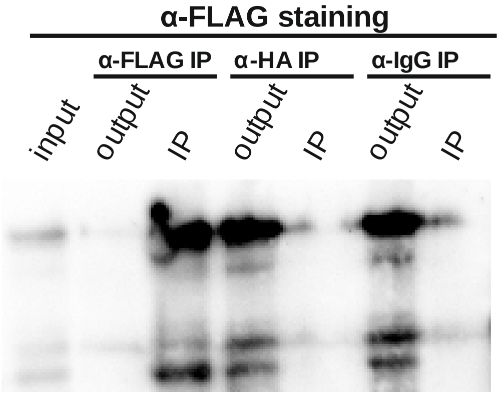

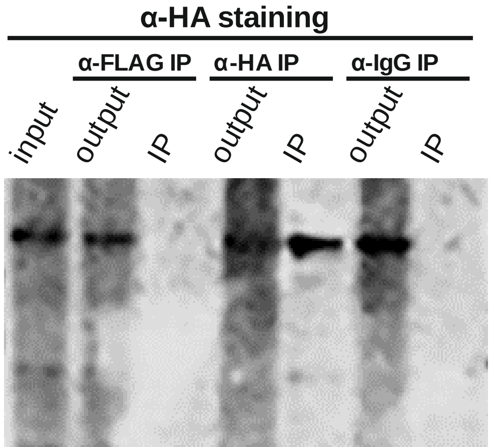

ciCTCF	'medium-3'		
	BD	BD-ci	ci-BD
AD	-	-	-
AD-ci	-	-	+
ci-AD	-	-	-

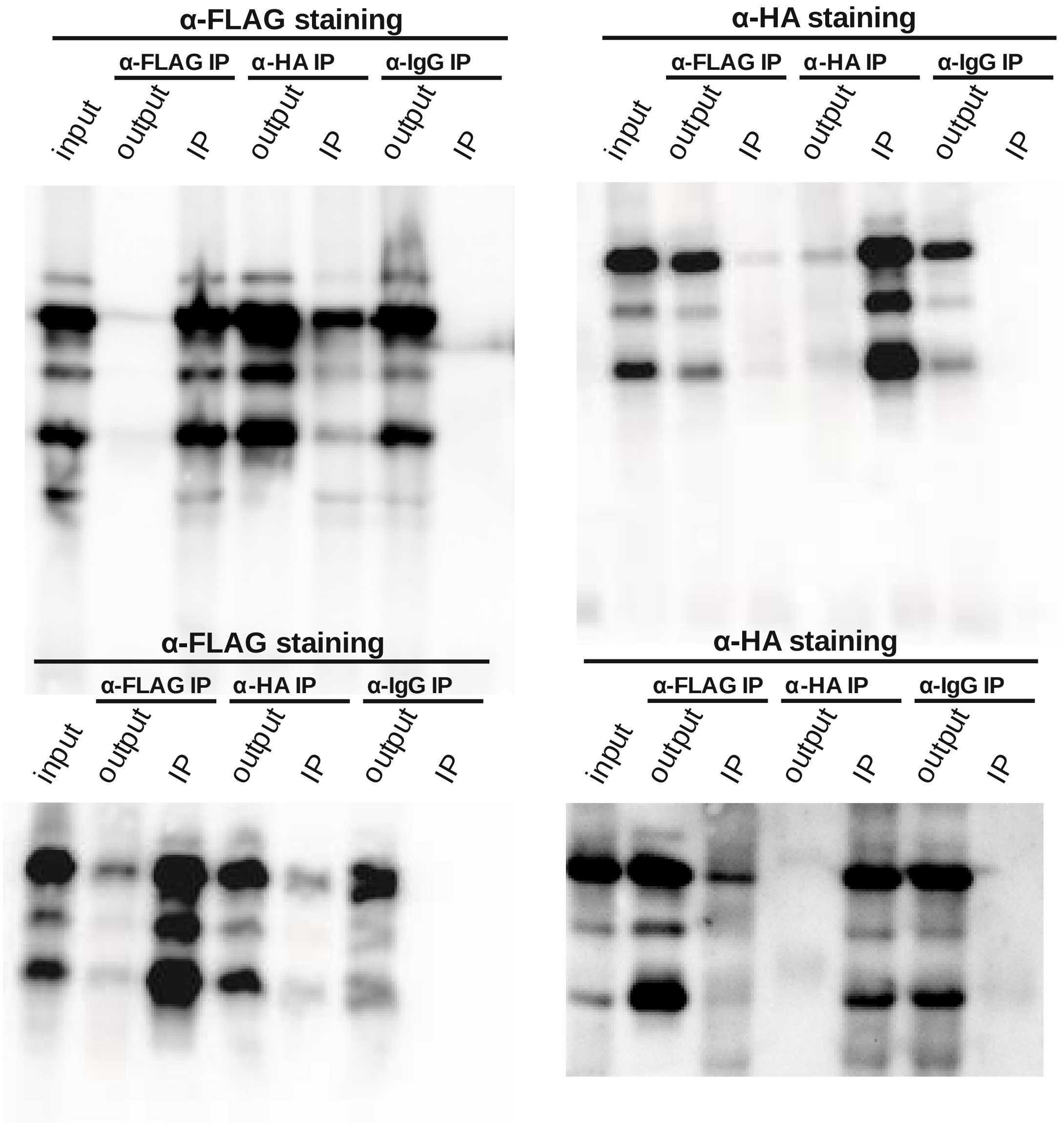

hsCTCF	'medium-3'		
	BD	BD-hs	hs-BD
AD	-	+	-
AD-hs	-	+	+
hs-AD	-	+	-

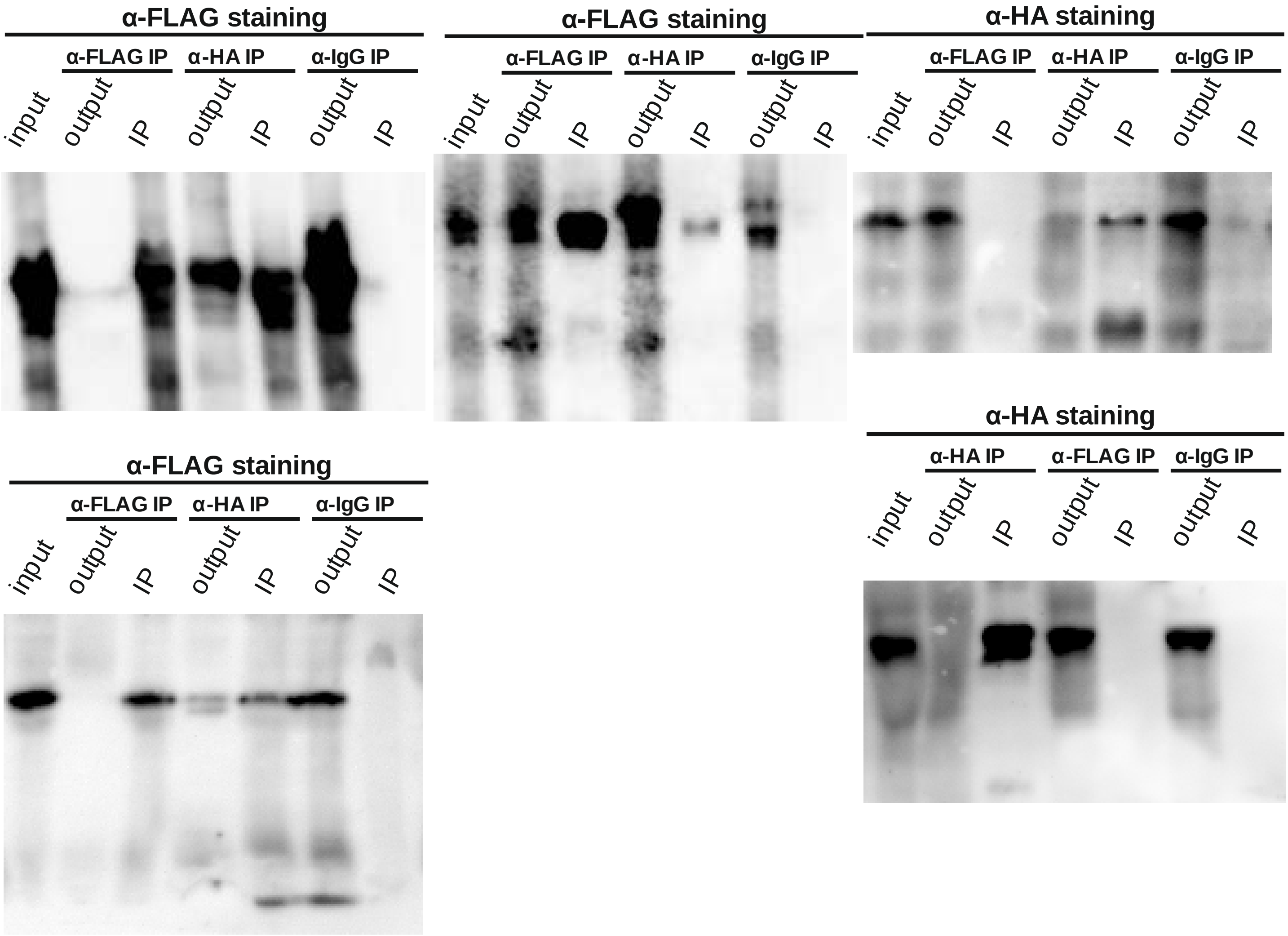

drCTCF	'medium-3'			'medium-3' + 2mM 3AT			'medium-3' + 5mM 3AT			'medium-3' + 15mM 3AT			'medium-3' + 20mM 3AT			'medium-3' + 25mM 3AT			'medium-3' + 50mM 3AT		
	BD	BD-dr	dr-BD	BD	BD-dr	dr-BD	BD	BD-dr	dr-BD	BD	BD-dr	dr-BD	BD	BD-dr	dr-BD	BD	BD-dr	dr-BD	BD	BD-dr	dr-BD
AD	-	+	-	-	+	-	-	+	-	-	+	-	-	+	-	-	+	-	-	+	-
AD-dr	-	+	-	-	+	-	-	+	-	-	+	-	-	+	-	-	+	-	-	+	-
dr-AD	-	+	-	-	+	-	-	+	-	-	+	-	-	+	-	-	+	-	-	+	-

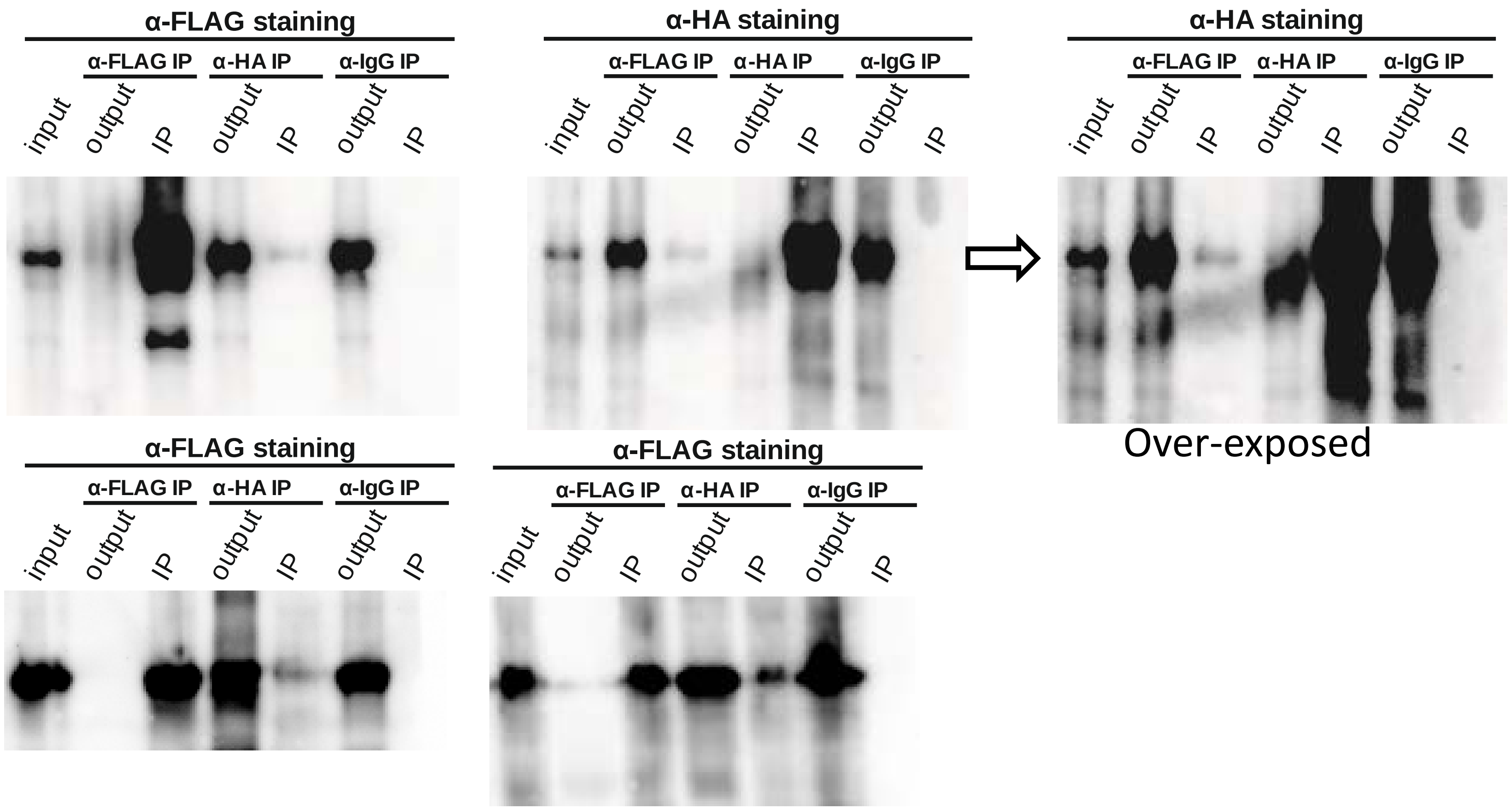

Table S3. The list of oligonucleotides used in work.

	**direct**	**reverse**
**amCTCF**	gatggatCCATGACAAGTAACGTAGCAG	GAGAaTTcTTAGGTAAAAATTTAATAATTTTTGTTTTGTC
**ciCTCF**	gaaagatctATGGCAGATGATGGAAAAGATAG	TTCGAATTCTTTTTAAACATGGTTGG
**cgCTCF**	aaaggatCCATGGACTCGCAACAGGAACTG	gtggaattcCCATGGATGTCAAACTTTGGAGC
**drCTCF**	aagaATTcCCCATGGAAGGGGGAC	ttctcgagCACCTTTCTTCTTGATTTTGGTTGG
**dpCTCF**	tagggatCCATGGCGGCCTTGAAGCAAG	acggaattcGTGACCTTTTTGGAGCCACC
**dvCTCF**	ttggatccATGTCAAAACGCACGCGCG	GGGaaTTCaCTTTGGAGGAGCGGGG
**dmCTCF**	CGGATCCATGCCAAGGAGGACA	ttctcgagCAGCTGCTCGGGCTGGTCGAAC
**hsCTCF**	GAGAATTCATGGAAGGTGATGCAGTCG	CTCACACTGGAATGTCTTCT
**skCTCF**	tagggatcCATGGAGACCAACCAACCTCAGAC	ggtgaaTTCTTCACTCTATTAAACACATGGTC
**spCTCF**	attggatccATGGATGAAAACACAGATCAACC	tcagaattCTTGGGCACCACATACCTGGTCTTC

FIGURES

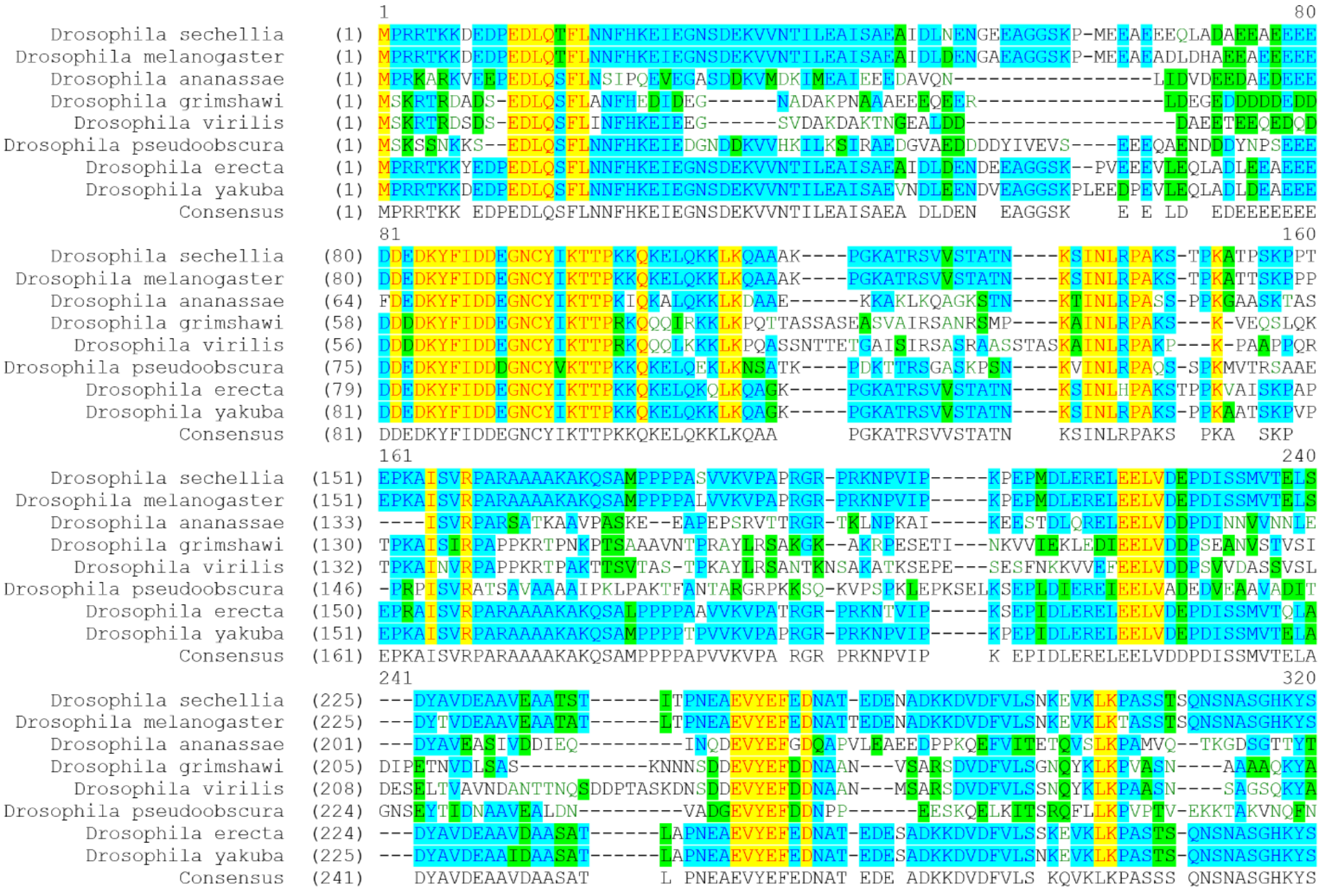

Figure S1. Multiple sequence alignment of CTCF N-terminal domain sequences from different *Drosophila* species. Dimerization region was mapped between 70-163 amino acids of dmCTCF.

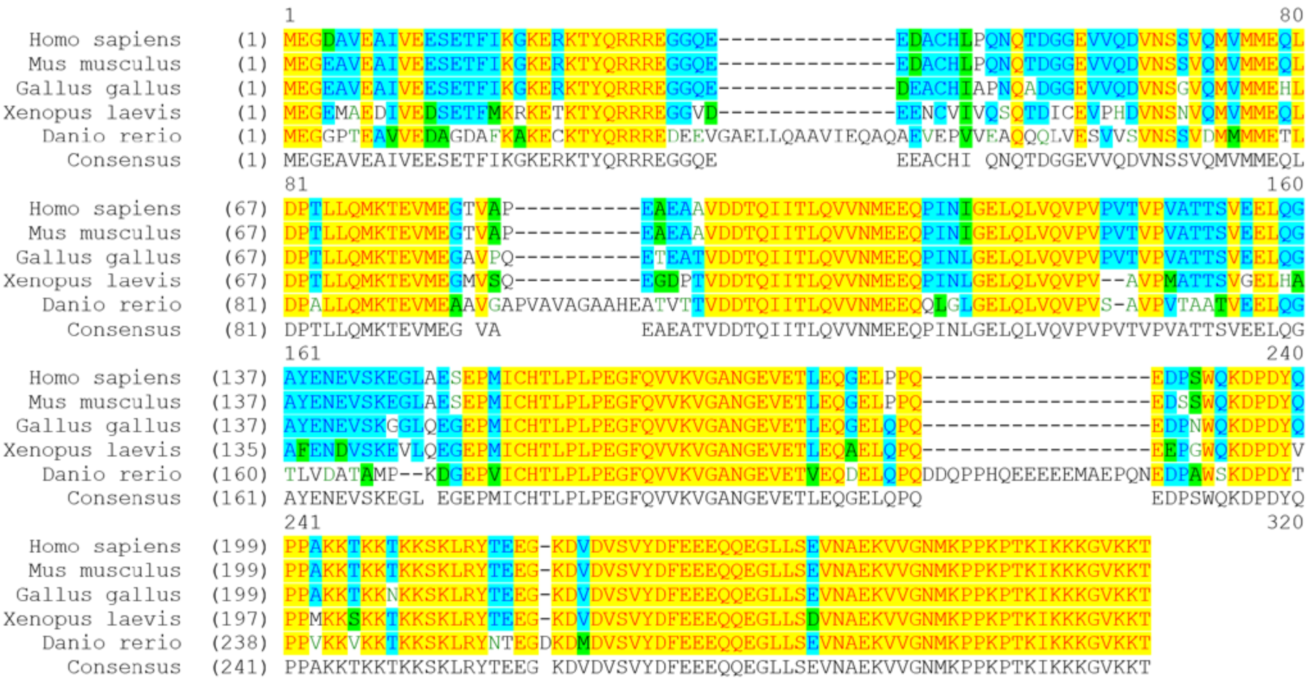

Figure S2. Multiple sequence alignment of the CTCF N-terminal domain sequences from various species of vertebrates.

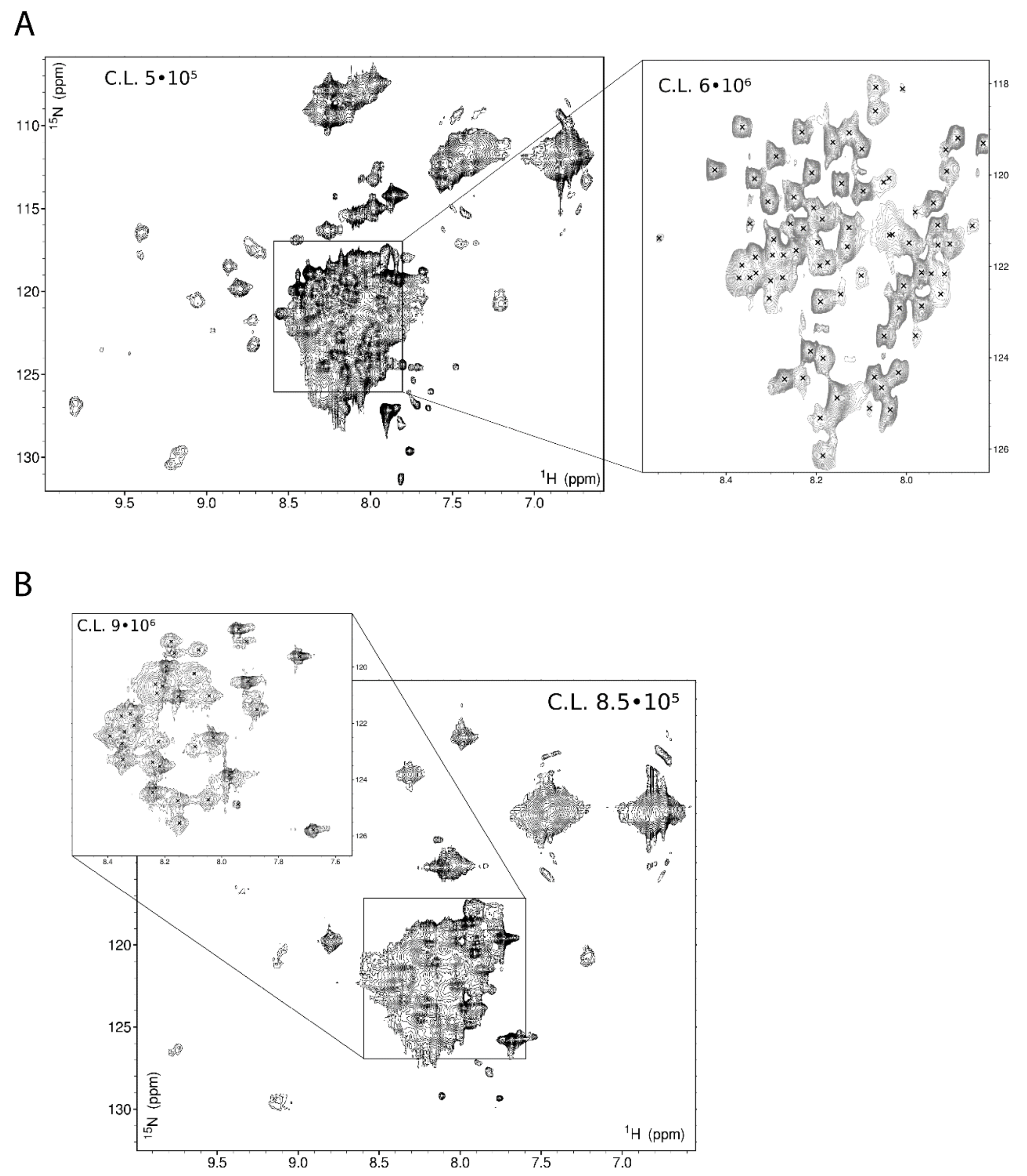

Figure S3. ^{15}N,^{1}H HSQC for ^{15}N-labelled dmCTCF NTD (A) and dvCTCF NTD (B). The spectra were recorded at the 50°C in 20 mM sodium phosphate (pH 6.5 for dmCTCF and 7.0 for dvCTCF) and 20 mM NaCl. C.L. stands for Contour Level.

In both cases, most amino acid residues have ^{1}H chemical shifts in the amide group from 7.8 to 8.5 ppm, which indicate that the corresponding residues belong to the unstructured part with very high mobility. Thus, about 100 individual signals can be detected in the disordered region for dmCTCF-NTD and about 60 for dvCTCF-NTD. At the same time there are at least 15 signals in ^{15}N,^{1}H HSQC of dmCTCF and 6 in ^{15}N,^{1}H HSQC of dvCTCF with ^{1}H chemical shift value in the range from 8.5 to 10 ppm, which represent residues located in structured regions. To improve spectra quality caused by fast relaxation we obtained ^{2}H,^{15}N-dvCTCF NTD sample and successfully refolded it using 8M urea, but resulting spectrum was exactly the same, without appearance of new peaks, so we conclude that some resonances from the structured region could not appear on the spectra due to the fast exchange of amide protons with water or conformational broadening.

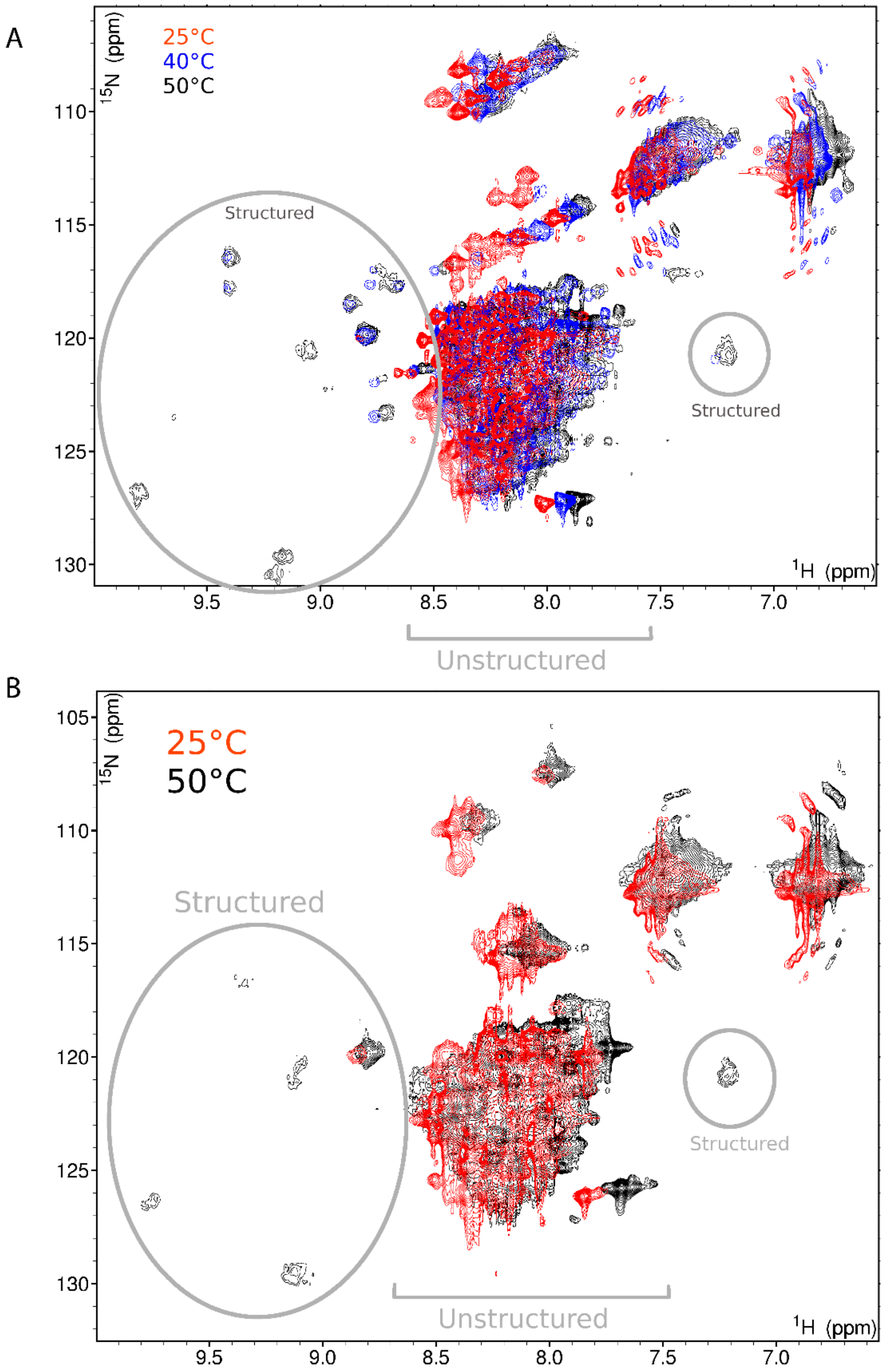

Figure S4. A) ^{15}N,^{1}H HSQC spectra of ^{15}N-labelled dmCTCF NTD at the temperatures 25°C (red), 40°C (blue), and 50°C (black). B) ^{15}N,^{1}H HSQC spectra for ^{15}N-labelled dvCTCF NTD at the temperatures 25°C (red) and 50°C (black). Signals corresponding to amide groups of residues placed in structured protein region are circled. The area of spectra with the signals of residues located in the unstructured part is indicated.

In both cases, at low temperature, only signals from residues located in disordered regions could be detected, while signals of residues from structured protein fragments undergo fast relaxation due to slow molecule tumbling. With increasing temperature and speed of molecular tumbling, the signals from the structured region became more intense.
